# Eldecalcitol prevented OVX-induced osteoporosis through inhibiting BMSCs senescence by regulating the SIRT1-Nrf2 signal

**DOI:** 10.3389/fphar.2023.1067085

**Published:** 2023-03-03

**Authors:** Yuying Kou, Xing Rong, Rong Tang, Yuan Zhang, Panpan Yang, Hongrui Liu, Wanli Ma, Minqi Li

**Affiliations:** ^1^ Department of Bone Metabolism, School and Hospital of Stomatology, Cheeloo College of Medicine, Shandong University and Shandong Key Laboratory of Oral Tissue Regeneration and Shandong Engineering Laboratory for Dental Materials and Oral Tissue Regeneration and Shandong Provincial Clinical Research Center for Oral Diseases, Jinan, China; ^2^ Center of Osteoporosis and Bone Mineral Research, Shandong University, Jinan, China; ^3^ Department of Traumatic Orthopedics, The Second Hospital of Shandong University, Jinan, China

**Keywords:** osteoporosis, eldecalcitol, BMSCs, senescence, SIRT1-Nrf2 signal

## Abstract

**Background:** Aging and oxidative stress are considered to be the proximal culprits of postmenopausal osteoporosis. Eldecalcitol (ED-71), a new active vitamin D derivative, has shown a good therapeutic effect on different types of osteoporosis, but the mechanism is unclear. This study focused on exploring whether ED-71 could prevent bone loss in postmenopausal osteoporosis by regulating the cell senescence of bone mesenchymal stem cells (BMSCs), and explaining its specific mechanism of action.

**Materials and methods:** An ovariectomized (OVX) rat model was established and 30 ng/kg ED-71 was administered orally once a day. The weight of rats was recorded regularly. Micro-computed tomography (CT) and histochemical staining were used to evaluate bone mass, histological parameters, and aging-related factors. Rat bone mesenchymal stem cells were extracted and cultivated *in vitro*. Aging cells were marked with senescence-associated β-gal (SA-β-gal) dyeing. The mRNA and protein levels of aging-related factors and SIRT1-Nrf2 signal were detected by RT-PCR, Western blot, and immunofluorescence staining. The reactive oxygen species (ROS) levels were detected by DCFH-DA staining.

**Results:** Compared with the Sham group, the bone volume of the ovariectomized group rats decreased while their weight increased significantly. ED-71 prevented bone loss and inhibited weight gain in ovariectomized rats. More importantly, although the expression of aging-related factors in the bone tissue increased in the ovariectomized group, the addition of ED-71 reversed changes in these factors. After extracting and *in vitro* culturing bone mesenchymal stem cells, the proportion of aging bone mesenchymal stem cells was higher in the ovariectomized group than in the Sham group, accompanied by a significant decrease in the osteogenic capacity. ED-71 significantly improved the bone mesenchymal stem cells senescence caused by ovariectomized. In addition, ED-71 increased the expression of SIRT1 and Nrf2 in ovariectomized rat bone mesenchymal stem cells. Inhibition of SIRT1 or Nrf2 decreased the inhibitory effect of ED-71 on bone mesenchymal stem cells senescence. ED-71 also showed a suppression effect on the reactive oxygen species level in bone mesenchymal stem cells.

**Conclusion:** Our results demonstrated that ED-71 could inhibit the cell senescence of bone mesenchymal stem cells in ovariectomized rats by regulating the SIRT1-Nrf2 signal, thereby preventing bone loss caused by osteoporosis.

## 1 Introduction

Osteoporosis, an increasingly severe social problem, is characterized by systemic damage to the bone mass, strength, and microstructure, increasing the tendency for fragility fracture (Rachner et al., 2011). Postmenopausal osteoporosis is characterized by insufficient estrogen secretion starting from the perimenopausal period, which leads to a more severe decrease in bone density and fracture risk in elderly women than in elderly men (Wei et al., 2021). During postmenopausal osteoporosis, osteoblasts and osteoclasts in bone tissue are affected and the balance of bone remodeling is destroyed, and a severe increase in bone absorption occurs, resulting in bone loss (Eriksen et al., 1990). However, recent studies have found that the inflammatory state caused by estrogen deficiency could damage the bone marrow environment and affect the physiological processes of cells (Weitzmann and Pacifici, 2006; Fischer and Haffner-Luntzer, 2022); among them, cell senescence has received increasing attention (Wu et al., 2020).

Cell senescence represents a permanent cell growth stagnation state (Salama et al., 2014), manifested by an arrested cell cycle, senescence-associated secretory phenotype (SASP), macromolecular injury and metabolic disorders (Sikora et al., 2021). Cell senescence is an important driving factor for aging and many diseases (Rossman et al., 2017; Sun et al., 2018; Song et al., 2020). The causes of cell senescence are considered to be aging, oxidative stress, accumulated damaged DNA molecules/mutations, and the shortening of the telomeres (Horvath and Raj, 2018; da Costa et al., 2016), but they aren’t completely clear. Studies have found that estrogen is closely related to cell senescence and age-related diseases. Estrogen is considered a key deciding factor in the aging of non-reproductive peripheral tissues, especially bone, skin, and brain (Emmerson and Hardman, 2012). Estrogen regulates the stemness and aging of bone mesenchymal stem cells (BMSCs) by inhibiting the ERβ-SATB2 pathway to prevent osteoporosis (Wu et al., 2018). Estrogen also regulates USP10 in osteoblasts and osteocytes, which accelerates P53 degradation, preventing cell senescence (Wei et al., 2021). A study has found that apoptosis and aging cells were increased in postmenopausal osteoporosis, and the expression levels of P53 and P16 is raised (Sui et al., 2016). Lack of P16 inhibits oxidative stress, osteocyte senescence, and osteoclastic bone resorption, stimulating osteogenesis and osteoblastic bone formation (Li et al., 2020). Therefore, the intervention of cell senescence might be an effective treatment for osteoporosis caused by the estrogen deficiency.

Adult stem cells are considered to be primarily responsible for cell function loss and senescence related to age (Yu et al., 2022). Mesenchymal stem cells (MSCs) originating from adult bone marrow stroma are the best-characterized mesoderm-derived stromal cells with multipotent differentiation capacity, which is related to the regeneration and stability of the tissue (Klimczak and Kozlowska, 2016). BMSCs, which mainly differentiate into osteoblasts and adipocytes (Kassem and Bianco, 2015), have the potential to treat a variety of diseases, including osteoporosis (OP), diabetes (DM), osteoarthritis (OA), myocardial infarction (MI), and Crohn’s disease (CD) (Liu et al., 2021). JAK suppression protects BMSCs and prevents them from aging, thereby preventing bone loss in ovariectomized (OVX) mice (Wu et al., 2020). Melatonin prevents estrogen deficiency-related bone loss by improving BMSCs resistance to cell senescence (Chen et al., 2022). Therefore, regulating BMSC senescence may be an important way to treat osteoporosis.

A study has shown that supplementation of vitamin D and calcium is recommended for patients with osteoporosis as a baseline treatment (Rachner et al., 2011). Vitamin D is synthesized in the skin or absorbed from the diet. In the liver, it is mainly transformed into 25-hydroxy-vitamin D by CYP2R1, and then it is transformed into 1, 25-dihydroxy-vitamin D, which has biological activity *via* CYP27B1 in the kidney (Goltzman, 2018). Active vitamin D combines with the vitamin D receptor (VDR), increasing the absorption of calcium in the intestine to maintain the calcium and phosphorus balance, or directly regulating bone metabolism by affecting the muscle and bones (Gunton et al., 2015; Anderson, 2017). It has also been found to play the role of anti-aging. 1, 25(OH)_2_D_3_ plays a role in preventing age-related osteoporosis by upregulating Ezh2 and inhibiting the senescence of BMSCs (Yang et al., 2020). However, few studies have explored the relationship between active vitamin D and cell senescence in postmenopausal osteoporosis. In recent years, the development and usage of active vitamin D analogs have become hot spots. Eldecalcitol (ED-71), a second-generation active vitamin D analog with a hydroxypropyloxy residue at the 2β position, has been approved in Japan for the clinical treatment of osteoporosis (Sakai et al., 2015). ED-71 provides a high combination with Vitamin D Binding Protein (DBP) (Kubodera et al., 2003) and a lower risk of persistent hypercalcemia (Matsumoto et al., 2005), which makes it a potential drug for osteoporosis treatment. Studies have found that ED-71 could significantly improve bone mineral density (BMD) in patients with postmenopausal osteoporosis (Iba et al., 2017), and reverse bone loss in OVX rats (de Freitas et al., 2011). ED-71 is more effective than Alfacalcidol in preventing vertebral and wrist fractures in patients with osteoporosis (Matsumoto et al., 2011). However, current research on the role of ED-71 in bone remodeling is mainly focused on the absorption of osteoclasts (Uchiyama et al., 2002; de Freitas et al., 2011). There is no research on whether it regulates cell senescence during the process of preventing osteoporosis.

Sirtuin1 (SIRT1) belongs to the family of NAD + -dependent deacetylases, and it is an important protective agent against oxidative stress and aging (Chen et al., 2020). Its activation prevents oxidative stress-induced endothelial senescence and dysfunction (Luo et al., 2021). In addition to histones, SIRT1 deacetylates non-histone substrates, including P53, FoxOs, PPAR-γ, and NF-κB (Nakagawa and Guarente, 2014). A study has found that 17β-estradiol could delay the cell senescence by upregulating SIRT1 (Han et al., 2013). Besides, Nuclear factor erythroid 2-related factor 2 (Nrf2) is a nuclear transcription factor that plays an indispensable role in inducing endogenous antioxidant enzymes against oxidative stress (Surh et al., 2008). Studies have shown that BMSCs senescence is accompanied by oxidative damage (Chen X. et al., 2019), and activation of Nrf2 is considered to inhibit cell senescence (Fang et al., 2018). SIRT1 can play the role of an upstream signal of Nrf2 (Xu et al., 2021; Lee et al., 2022). Studies have examined the relationship between vitamin D with SIRT1 and Nrf2. Vitamin D increases the osteogenic potential of BMSCs by raising SIRT1 (Borojević et al., 2022). Vitamin D is also found to regulate Nrf2 to inhibit oxidative stress and DNA damage in order to play a role in delaying senescence (Chen L. et al., 2019). These findings have increased our interest in the relationship between ED-71 and SIRT1-Nrf2 signal.

In this study, we explored the role of cell senescence during the occurrence of postmenopausal osteoporosis by establishing an OVX rat model. Next, we explored whether ED-71 improved the bone loss caused by OVX by regulating the senescence of BMSCs and explained its specific mechanism of action. Our research proposed a new mechanism of ED-71 to treat osteoporosis and provided a new idea for its clinical applications.

## 2 Materials and Methods

### 2.1 Animals and reagents

Twenty-four female Wistar rats were purchased from Jinan Pengyue Experimental Animal Breeding Co., Ltd. (Shandong, China). All animal experiments were approved by the Institutional Animal Care and Use Committee, School and Hospital of Stomatology, Shandong University (No. 20210912).

ED-71 was purchased from Chugai Pharmaceutical Co., Ltd. According to the manufacturer’s instructions, it was dissolved in absolute ethanol for storage. Before use, the solution was diluted to the corresponding concentration. The Anti-Osterix antibody (ab209484), Anti-P16 antibody (ab54210) and secondary antibodies (ab6721, ab102448, ab150081, and ab150119) were purchased from Abcam (Cambridge, United Kingdom). The Anti-Runx2 antibody (20700-1-AP), Anti-P53 antibody (60283-2-Ig), Anti-GAPDH antibody (10494-1-AP), Anti-SIRT1 antibody (60303-1-Ig) and Anti-Nrf2 antibody (16396-1-AP) were purchased from Proteintech (Chicago, United States). The Anti-β-gal antibody (bs-4631R) and Anti-Osteocalcin (OCN) antibody (bs-4917R) was purchased from Bioss (Beijing, China). The Anti-P16 antibody (A0262) was purchased from ABclonal (Wuhan, China). EX-527 (HY-15452) and ML-385 (HY-100523) were purchased from MedChemExpress (New Jersey, United States).

### 2.2 Establishment of an ovariectomized (OVX) rat model

Eight-week-old female Wistar rats were used to establish an OVX model. All animals were randomly divided into the following three groups (*n* = 8): Sham group, OVX group and OVX + ED-71 group. Further, 1% pentobarbital sodium was used to anesthetize rats. After performing an incision on the back and separating the subcutaneous tissue and muscles, the oviducts were ligated and the ovaries were removed. Then the wound was sutured and antibiotics were provided for 3 days after the operation. ED-71 was given *via* the oral route at a concentration of 30 ng/kg once a day. The weight of rats was monitored weekly. After 8 weeks, part of the rats were euthanized through excessive anesthesia. The fresh bone tissues of rats were harvested for the isolation of BMSCs or stored at −80°C. Other rats were fixed with 4% paraformaldehyde by cardiac perfusion. The femur was separated and decalcified in EDTA-2Na solution at 4°C. After dehydration in an ethanol gradient and transparent in xylene, the samples were embedded in paraffin and continuously cut into 5 μm thick slices.

### 2.3 Micro-computed tomography (CT) scan

After fixation with 4% paraformaldehyde by cardiac perfusion, rat femurs were separated. Then, these tissues were scanned at a resolution of 14.8 μm, a voltage of 70 kvp, and a current of 200 mA. The three-dimensional images were reconstructed with a micro-CT analysis system (Scanco Medical, Switzerland). The data were obtained from the region of interest (ROI), which was along the long axis of the distal femur.

### 2.4 Hematoxylin and eosin (HE) staining

The paraffin sections were immersed in xylene for dewaxing and hydrated in descending gradient alcohol. Next, the sections were stained with hematoxylin for 15 min and washed with distilled water. Then they were stained with eosin for 7 min and washed again. Finally, sections were dehydrated and mounted. An optical microscope (Olympus BX-53, Tokyo, Japan) was used to observe and obtain the digital image. Image pro Plus 6.0 (IPP 6.0) software (Media Cybernetics, Silver Spring, MD, United States) was used for the quantitative analysis.

### 2.5 Masson staining

Masson trichrome staining was used to identify the regenerated ossification. After being dewaxed and hydrated, slices were stained with hematoxylin for 10 min and washed with distilled water. Next, the slices were immersed in a Ponceau S acid fuchsin solution for 7 min and differentiated in a phosphomolybdic acid solution for 4 min. Then they were moved to the aniline blue solution directly and stained for 1 min. Finally, the slices were washed, dehydrated, and mounted. Stained sections were observed with an optical microscope (Olympus BX-53, Tokyo, Japan) and digital images were obtained.

### 2.6 Immunohistochemical staining

After being immersed in xylene and hydrated in alcohol, the sections were treated with 0.3% hydrogen peroxide for endogenous peroxidase inhibition, and then treated with 1% bovine serum albumin (BSA) in PBS for 20 min for non-specific staining blocking. They were incubated overnight with primary antibodies at 4°C. After washing with PBS, they were incubated with the secondary antibody for 1 h at room temperature. Finally, visualization was achieved by 3, 3′-diaminobenzidine tetrahydrochloride (DAB). Counterstaining with methyl green was performed for all sections. An optical microscope (Olympus BX-53, Tokyo, Japan) was used to observe and take photographs. The Image pro Plus 6.0 (IPP 6.0) software was used to analyze the positive expression in all sections (optical density, OD).

### 2.7 Extraction and culture of BMSCs

After 8 weeks of model establishment, the rat tibia and femur were separated to isolate BMSCs. They were dissected in a sterile manner and washed with PBS containing 5% penicillin and streptomycin. Then the proximal and distal ends were removed so that the bone marrow was exposed. The bone marrow was rinsed by a syringe with the *α*-modified Eagle’s medium (*α*-MEM) containing 20% fetal bovine serum (FBS). After allowing it to stand for 5–7 days, the medium was replaced. When the adherent cells reached 80%–90% confluence, they were digested with 0.25% trypsin-ethylene diamine tetra acetic acid. The third to fifth-generation cells were used.

### 2.8 Alkaline phosphatase (ALP) staining and alizarin red dyeing

The BMSCs were cultured in an osteogenic induction medium containing 0.1 μM dexamethasone, 10 mM β-glycerophosphate, and 0.05 mM ascorbic acid. The medium was replaced every 3 days. ALP staining was performed after osteogenic induction for 7 days. After being fixed for 20 min with 4% paraformaldehyde, BMSCs were incubated with ALP solution for 25 min and washed with PBS. Alizarin red dyeing was performed after osteogenic induction for 21 days. The BMSCs were fixed with 4% paraformaldehyde and stained with 1% alizarin red staining solution (G1452, Solarbio, China). The PBS solution was used for washing. Finally, ALP-positive osteoblasts and calcium nodules were observed with an optical microscope (Olympus BX-53, Tokyo, Japan). Image pro Plus 6.0 (IPP 6.0) software was used to analyze the ALP staining positive rate. Alizarin red was isolated with cetylpyridinium chloride and detected using a spectrophotometer at 450 nm.

### 2.9 Oil red O staining

BMSCs were cultured in an adipogenic induction medium. According to the instructions of the reagent, the cells were cultivated alternately with induction liquid A and induction liquid B for 21 days. The BMSCs were fixed with 4% paraformaldehyde and stained with the Oil Red O Solution (OILR-10001, OriCell, China). The formation of lipid droplets was observed by an optical microscope (Olympus BX-53, Tokyo, Japan).

### 2.10 Senescence β-galactosidase (β-gal) staining

Aging cells were stained with the Senescence β-Galactosidase Staining Kit (C0602, Beyotime, China). The cells were fixed for 15 min with a fixed solution and then washed 3 times with PBS. They were then incubated at 37°C overnight in the working dyeing fluid. Aging cells were observed under an optical microscope (Olympus BX-53, Tokyo, Japan), and the number of β-gal positive cells was counted.

### 2.11 Real-time polymerase chain reaction (PCR) analysis

The total RNA of tissues or cells was extracted by the RNAex Pro Reagent (Accurate Biology, Hunan, China), and cDNA was obtained by the Evo M-MLV reverse transcription kit (Accurate Biology, Hunan, China). Real-time PCR was performed with the SYBR Green PCR kit (Accurate Biology, Hunan, China). The relative expression levels of P16, P53, SIRT1, and Nrf2 were quantified using the 2^−ΔΔCT^ method, and normalized with the GAPDH level. All quantitative real-time PCRs were performed using the Roche Light Cycler 96 Real-time PCR system (Roche, Sussex, United Kingdom), and all samples were run in triplicate. The primer sequences are shown in Table 1.

**TABLE 1:** Specific primers for control and target genes.

Gene	Forward	Reverse
P16	5′-TGC​GGT​ATT​TGC​GGT​ATC​TAC​TCT​C -3′	5′-GGC​CTA​ACT​TAG​CGC​TGC​TTT​G-3′
P53	5′-GCC​ATC​TAC​AAG​AAG​TCA​CAA​CAC-3′	5′-TGT​CGT​CCA​GTA​CTC​AGC​ATA​C-3′
SIRT1	5′-TGA​CGC​CTT​ATC​CTC​TAG​TTC​CT-3′	5′-TCA​GCA​TCA​TCT​TCC​AAG​CCA​TT-3′
Nrf2	5′-TTA​AGC​AGC​ATA​CAG​CAG​GAC​AT-3′	5′-GGA​CAG​TGG​TAG​TCT​CAG​CCT-3′
GAPDH	5′-GAC​ATG​CCG​CCT​GGA​GAA​AC-3′	5′-AGC​CCA​GGA​TGC​CCT​TTA​GT-3′

### 2.12 Western blot analysis

The proteins of tissues and cells were extracted with RIPA Lysis Buffer (Cwbio, Beijing, China) mixed with protease and phosphatase inhibitors at a ratio of 98:1:1. The protein concentration was measured by the BCA protein assay (Beyotime, Beijing, China). Then the proteins were diluted in 1/4 volume of 5×SDS loading buffer and heated at 97°C for 5 min. Equal amounts of protein were separated in a 10% SDS-PAGE and transferred onto a PVDF membrane. They were incubated overnight with the primary antibody at 4°C, and then incubated with the secondary antibody at room temperature for 1 h. Finally, the enhanced chemiluminescence reagent (B500024, Proteintech Group, United States) and ECL detection system (Amersham Imager 600, General Electric Company, United States) were used to measure the immunoreactive bands. Each experiment was performed in triplicate.

### 2.13 Immunofluorescence staining

BMSCs were fixed with 4% paraformaldehyde, permeabilized with 0.5% Triton X-100, and blocked with 5% BSA in PBS. Then they were incubated with the primary antibody at 4°C overnight, and with the secondary antibody for 1 h at room temperature. The nuclei were stained by incubation with DAPI for 5 min. Finally, the cells were observed under a fluorescent microscope (DMi8 automated, Leica Microsystems CMS GmbH, Germany). The relative fluorescence intensity was measured by Image J software.

### 2.14 Measurement of the intracellular reactive oxygen species (ROS) level

Changes in the ROS level were detected by the ROS Assay Kit (Beyotime, China). After adding 200 μm H_2_O_2_ for 30 min, BMSCs were stained with 10 μM DCFH-DA for 30 min at 37°C. Then, they were washed with *α*-MEM without fetal bovine serum 3 times. Images were captured with a fluorescence microscope (DMi8 automated, Leica Microsystems CMS GmbH, Germany). Image J software was used to measure the relative fluorescence intensity.

### 2.15 Statistical analysis

All values were expressed as mean ± standard deviation (SD). Statistical analysis was performed using GraphPad Prism six software (San Diego, California, United States). The difference between the two groups was assessed by the Student’s t test, and one-way ANOVA was used for comparing multiple groups, with Fisher’s least significant difference (LSD) test for multiple comparisons. *p* < 0.05 was considered statistically significant.

## 3 Results

### 3.1 ED-71 prevented OVX-induced osteoporosis *in vivo*

**FIGURE 1:**
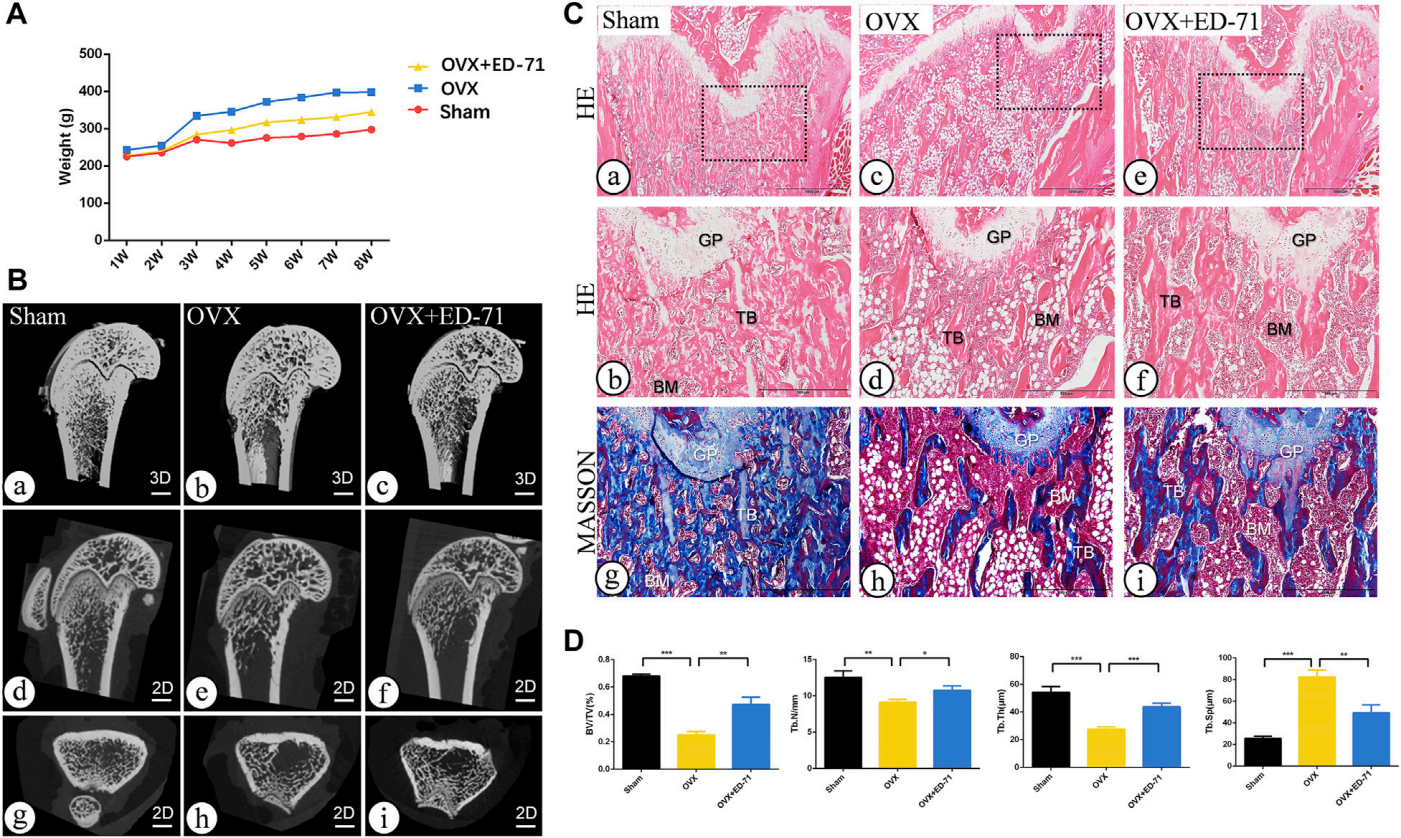
The effect of ED-71 preventing the bone loss of OVX rat. **(A)** The curve of the weigh change of rats in Sham, OVX, and OVX + ED-71 groups. **(B)** The representative 2D and 3D Micro-CT image of rat femur. Bar, 1 mm. **(C)** The HE staining and Masson staining images of rat femur. Bar, 1,000 μm or 500 μm. **(D)** Statistical analysis for the bone volume fraction (BV/TV), trabecular number, trabecular thickness and trabecular separation. All experiments were carried out at least 3 times, and data are expressed as mean ± SD. *p < 0.05. **p < 0.01. ***p < 0.001. GP, Growth Plate. TB, trabecular bone. BM, bone marrow.

**FIGURE 2:**
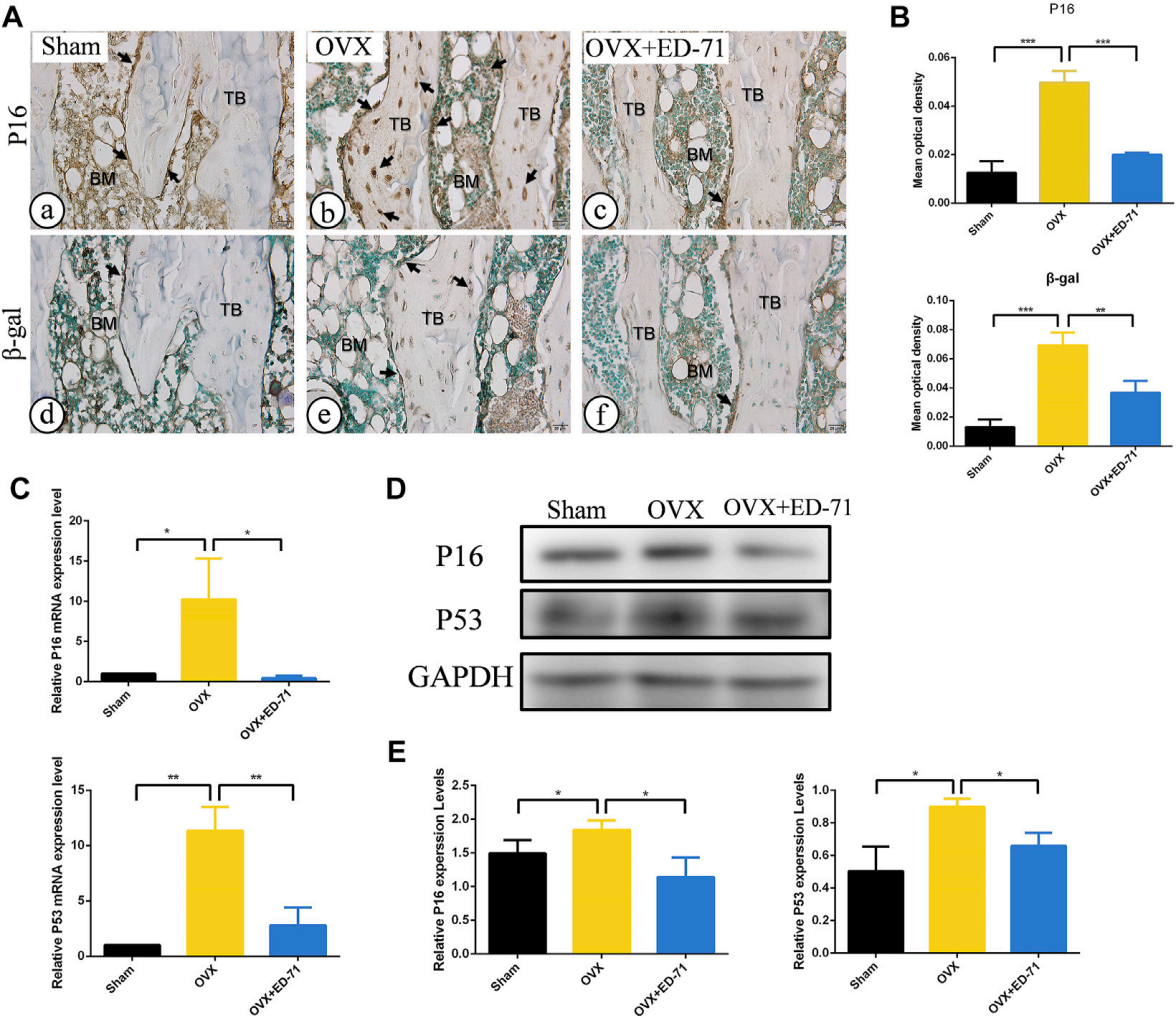
The effect of ED-71 on cell senescence in Sham, OVX, and OVX + ED-71 group. **(A)** The immunohistochemical staining image of P16 and β-gal in rat femur. Bar, 20 μm. **(B)** Statistical analysis of the immunohistochemistry results. **(C)** The mRNA expression of P16 and P53 in bone tissue were detected by RT-PCR. **(D)** The protein level of P16 and P53 in bone tissue were analyzed by Western blot. **(E)** The statistical analysis of Western blot results. All experiments were carried out at least 3 times, and data are expressed as mean ± SD. *p < 0.05. **p < 0.01. ***p < 0.001. TB, trabecular bone. BM, bone marrow.

The OVX rat model was established to explore the preventive effect of ED-71 on postmenopausal osteoporosis. The weight change curve showed that the weight of all rats was increased with age. Compared to the Sham group, weight of OVX rats was increased significantly, while ED-71 reversed the weight gain caused by OVX (Figure 1A). Micro-CT observed the 2D- and 3D-images of the femur, and it was clearly noted that the bone volume in OVX rats decreased compared to the Sham group, and this decrease in bone volume was improved in the OVX + ED-71 group (Figure 1B). HE staining showed that compared to the Sham group, the femoral bone volume and the number of trabecular bones were significantly reduced in the OVX group, accompanied by the thickness became narrow and the distribution became discrete and irregular. There was also a significant increase in lipid droplets in the bone marrow cavity. In the OVX + ED-71 group, the number and thickness of trabecular bones increased, and the separation of the trabecular bone and lipid droplets decreased (Figure 1C, D). Masson staining showed that the regeneration of new bone in the OVX group was significantly reduced compared to the Sham group, while it was increased in the OVX + ED-71 group (Figure 1C). In addition, the expression levels of the aging-related factors P16 and β-gal in the femur of OVX rats were significantly upregulated, while ED-71 reduced this increase (Figures 2A, B). The mRNA expression levels of P16 and P53 in the bone tissue of rats in the OVX group were higher than in the Sham and OVX + ED-71 groups (Figure 2C). The protein levels of P16 and P53 in bone tissues also showed the same trend (Figures 2D, E), indicating that ED-71 inhibited the senescence of bone tissue cells under the OVX state. These results suggested that ED-71 inhibited OVX-induced weight gain, bone loss, and cell senescence.

### 3.2 OVX accelerated the senescence of rat BMSCs

**FIGURE 3:**
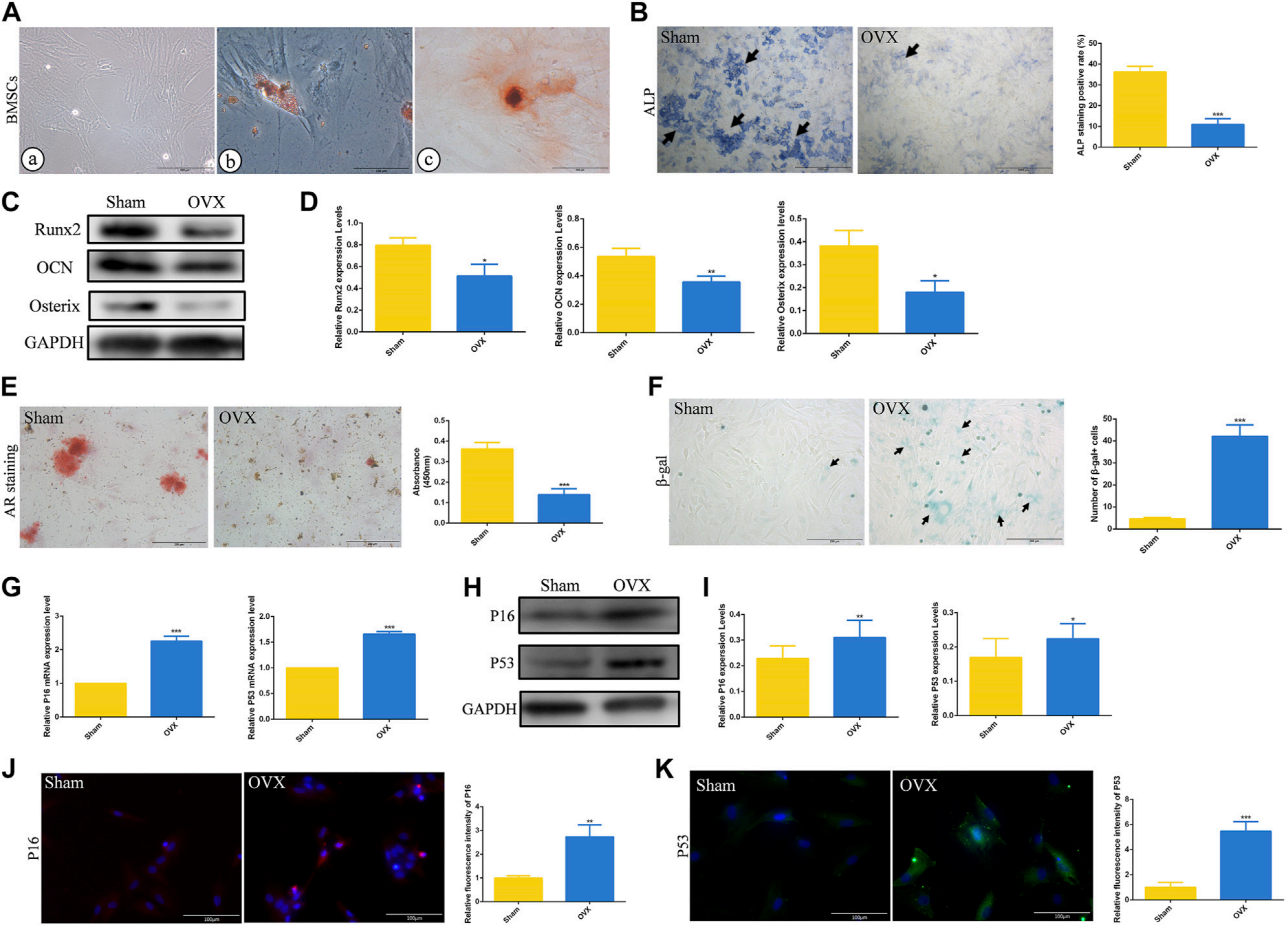
The osteogenesis ability and senescence level of rat BMSCs. **(A)** The morphology of rat BMSCs primary cells under the optical microscope, and the oil red O and alizarin red staining of BMSCs. Bar, 200 μm or 100 μm. **(B)** ALP staining of rat BMSCs in Sham and OVX group. Bar, 1,000 μm. **(C)** The protein level of Runx2, OCN, and Osterix in BMSCs after osteogenic induction were analyzed by Western blot. **(D)** The statistical analysis of Western blot results. **(E)** Alizarin red staining of rat BMSCs in Sham and OVX group. Bar, 200 μm. **(F)** The cytochemical staining of senescence-associated β-gal (SA-β-gal) and the statistical analysis. Bar, 200 μm. **(G)** The mRNA expression of P16, P19, and P53 in BMSCs were detected by RT-PCR. **(H)** The protein level of P16 and P53 in BMSCs were analyzed by Western blot. **(I)** The statistical analysis of Western blot results. **(J)** The immunofluorescence staining of P16 in rat BMSCs and the statistical analysis. Bar, 100 μm. **(K)** The immunofluorescence staining of P53 in rat BMSCs and the statistical analysis. Bar, 100 μm. All experiments were carried out at least 3 times, and data are expressed as mean ± SD. *p < 0.05. **p < 0.01. ***p < 0.001.

To further explore the role of cell senescence during ED-71 preventing osteoporosis, BMSCs from rats in the Sham and OVX groups were extracted and cultivated *in vitro*. Under the optical microscope, BMSCs showed a polygonal shape (Figure 3A). At the same time, BMSCs showed good potential for osteogenesis and adipogenesis *in vitro* (Figure 3A). ALP staining showed that after 7 days of osteogenic induction, ALP-positive osteoblasts in the OVX group were significantly reduced than that in the Sham group, prompting that the osteogenesis ability of OVX rat BMSCs was reduced (Figure 3B). Western blot also showed that the expressions of Runx2, OCN, and Osterix in BMSCs after 14 days of osteogenic induction were significantly decreased in the OVX group (Figures 3C, D). Alizarin red staining showed that the mineralization ability of BMSCs in OVX group was significantly decreased compared with that in Sham group (Figure 3E). More importantly, the OVX group had more β-gal positive cells than the Sham group (Figure 3F). Higher mRNA expression levels of P16, and P53 were observed in the OVX group (Figure 3G). The protein levels of P16 and P53 in the OVX group also increased (Figures 3H, I). Immunofluorescence staining of P16 and P53 showed the same trend (Figures 3J, K). These results suggested that aging cells in BMSCs of OVX rats were significantly increased compared to those in BMSCs of Sham rats.

### 3.3 ED-71 improved the cell senescence of OVX rat BMSCs

**FIGURE 4:**
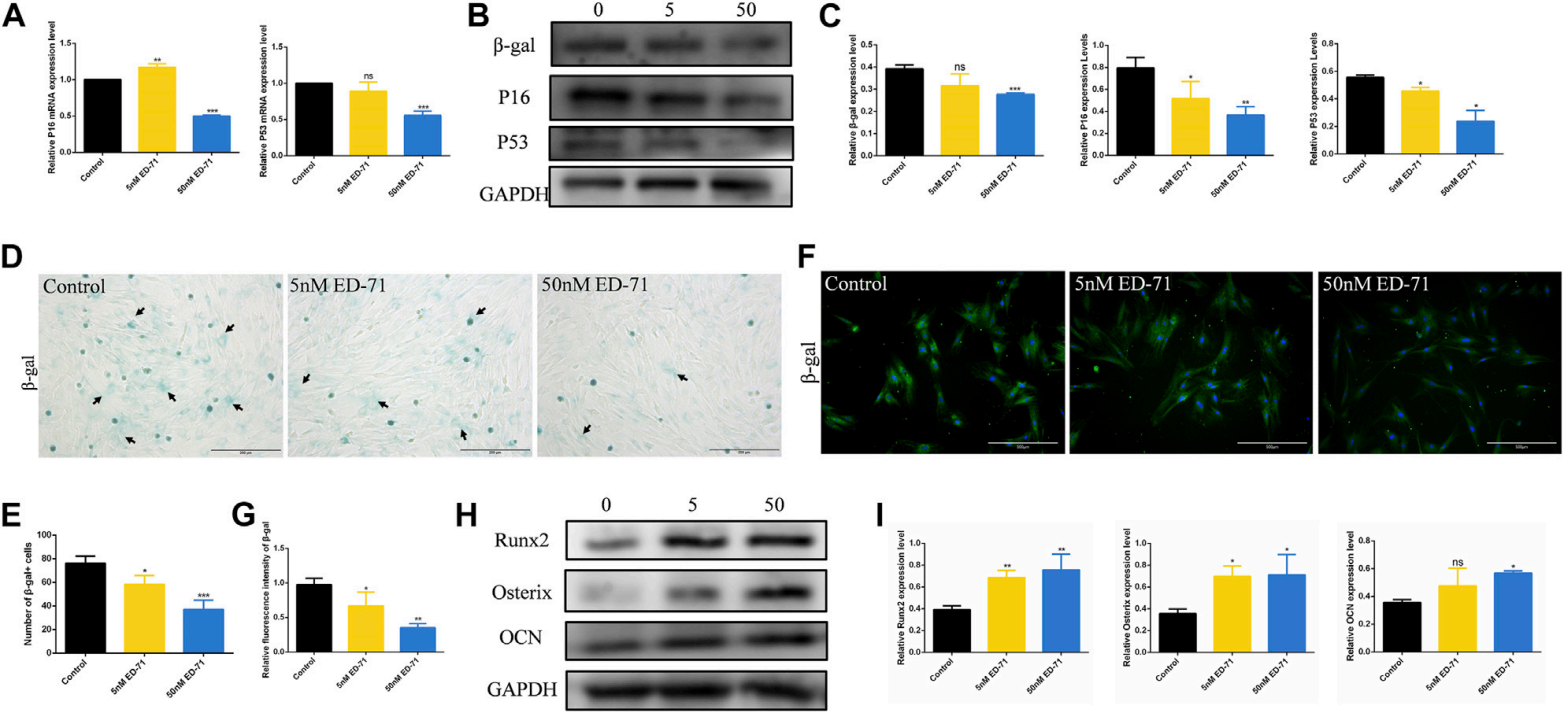
The effect of ED-71 on senescence of BMSCs. **(A)** The mRNA expression of P16 and P53 in BMSCs with or without the existence of ED-71 were detected by RT-PCR. **(B)** The protein level of P16, P53, and β-gal in BMSCs were analyzed by Western blot. **(C)** The statistical analysis of Western blot results. **(D)** The cytochemical staining of SA-β-gal. Bar, 200 μm. **(E)** The statistical analysis of β-gal staining. **(F)** The immunofluorescence staining of β-gal. Bar, 500 μm. **(G)** The statistical analysis of the fluorescence intensity. **(H)** The protein level of Runx2, Osterix, and OCN in BMSCs after osteogenic induction were analyzed by Western blot. **(I)** The statistical analysis of Western blot results. All experiments were carried out at least 3 times, and data are expressed as mean ± SD. *p < 0.05. **p < 0.01. ***p < 0.001.

After adding 50 nM ED-71 to OVX rat BMSCs, RT-PCR showed that ED-71 could downregulate the mRNA expression levels of P16 and P53 in BMSCs (Figure 4A). Western blot also showed that the protein levels of β-gal, P16, and P53 in BMSCs were suppressed by the addition of ED-71 (Figures 4B, C). The results of β-gal staining showed that the number of aging cells was gradually decreased in the control, 5 nM ED-71, and 50 nM ED-71 groups, indicating that ED-71 significantly improved the cell senescence of rat BMSCs induced by OVX (Figures 4D, E). With respect to immunofluorescence staining of β-gal, the positive expression of β-gal was significantly reduced after adding 5 nM and 50 nM ED-71 (Figures 4F, G). Besides, the results of Western blot showed that ED-71 significantly increased the protein expression of Runx2, Osterix and OCN in BMSCs after 14 days of osteogenic induction, suggesting that ED-71 promoted the osteogenic differentiation of BMSCs (Figures 4H, I). Therefore, ED-71 could significantly inhibit cell senescence and promote osteogenic differentiation.

### 3.4 ED-71 upregulated the SIRT1-Nrf2 signal in OVX rat BMSCs

**FIGURE 5:**
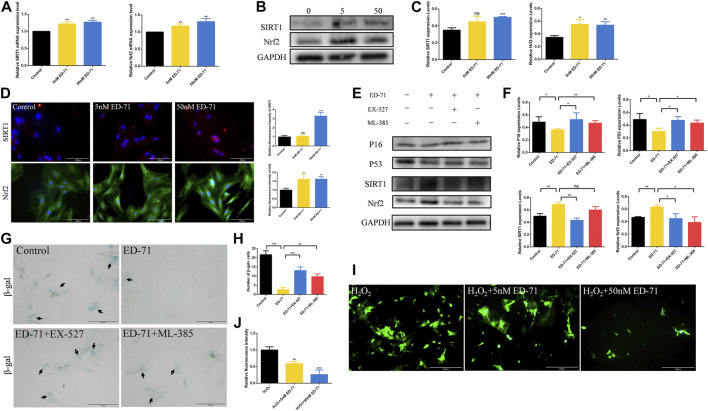
The effect of ED-71 on the SIRT1-NRF2 signal. **(A)** The mRNA expression of SIRT1 and Nrf2 in BMSCs with or without the existence of ED-71 were detected by RT-PCR. **(B)** The protein level of SIRT1 and Nrf2 in BMSCs were analyzed by Western blot. **(C)** The statistical analysis of Western blot results. **(D)** The immunofluorescence staining of SIRT1 and Nrf2 and the statistical analysis of the fluorescence intensity. Bar, 100 or 200 μm. **(E)** The protein level of P16, P53, SIRT1, and Nrf2 in BMSCs were analyzed by Western blot. **(F)** The statistical analysis of Western blot results. **(G)** The cytochemical staining of SA-β-gal. Bar, 200 μm. **(H)** The statistical analysis of β-gal staining. **(I)** The ROS levels in BMSCs was detected by DCFH-DA staining. Bar, 500 μm. **(J)** The statistical analysis of DCFH-DA staining. All experiments were carried out at least 3 times, and data are expressed as mean ± SD. *p < 0.05. **p < 0.01. ***p < 0.001.

Next, we further explored the specific mechanism of ED-71 for inhibiting the cell senescence of OVX rat BMSCs. RT-PCR showed that after adding 5 nM or 50 nM ED-71, the mRNA expression levels of SIRT1 and Nrf2 were increased significantly in BMSCs (Figure 5A). The results of Western blot showed the same trend (Figures 5B, C). SIRT1 and Nrf2 also showed stronger fluorescence in the ED-71 group, suggesting that their expression was upregulated (Figure 5D). To prove our discovery, SIRT1 inhibitor EX-527 and Nrf2 inhibitor ML-385 were used to block the expression of SIRT1 or Nrf2 respectively. Western blot showed that the addition of EX-527 significantly inhibited the promotion effect of ED-71 on the expression of SIRT1, and at the same time, it also inhibited the protein level of Nrf2. The use of ML-385 blocked the promotion effect of ED-71 on Nrf2. Simultaneously, the additions of EX-527 and ML-385 reversed the inhibitory effect of ED-71 on the expression of P16 and P53 (Figures 5E, F). The results of β-gal staining showed that the addition of ED-71 reduced the number of aging cells, while the use of SIRT1 and Nrf2 inhibitors reversed this effect (Figures 5G, H). In addition, after adding 200 μM H_2_O_2_ for 30 min, obvious accumulation of ROS occurred in BMSCs, and this ROS accumulation was reduced by the use of ED-71 (Figures 5I, J). These results proved that ED-71 could inhibit the cell senescence through the SIRT1-Nrf2 signal, and it could also protect the BMSCs from damage caused by oxidative stress.

## 4 Discussion

**FIGURE 6:**
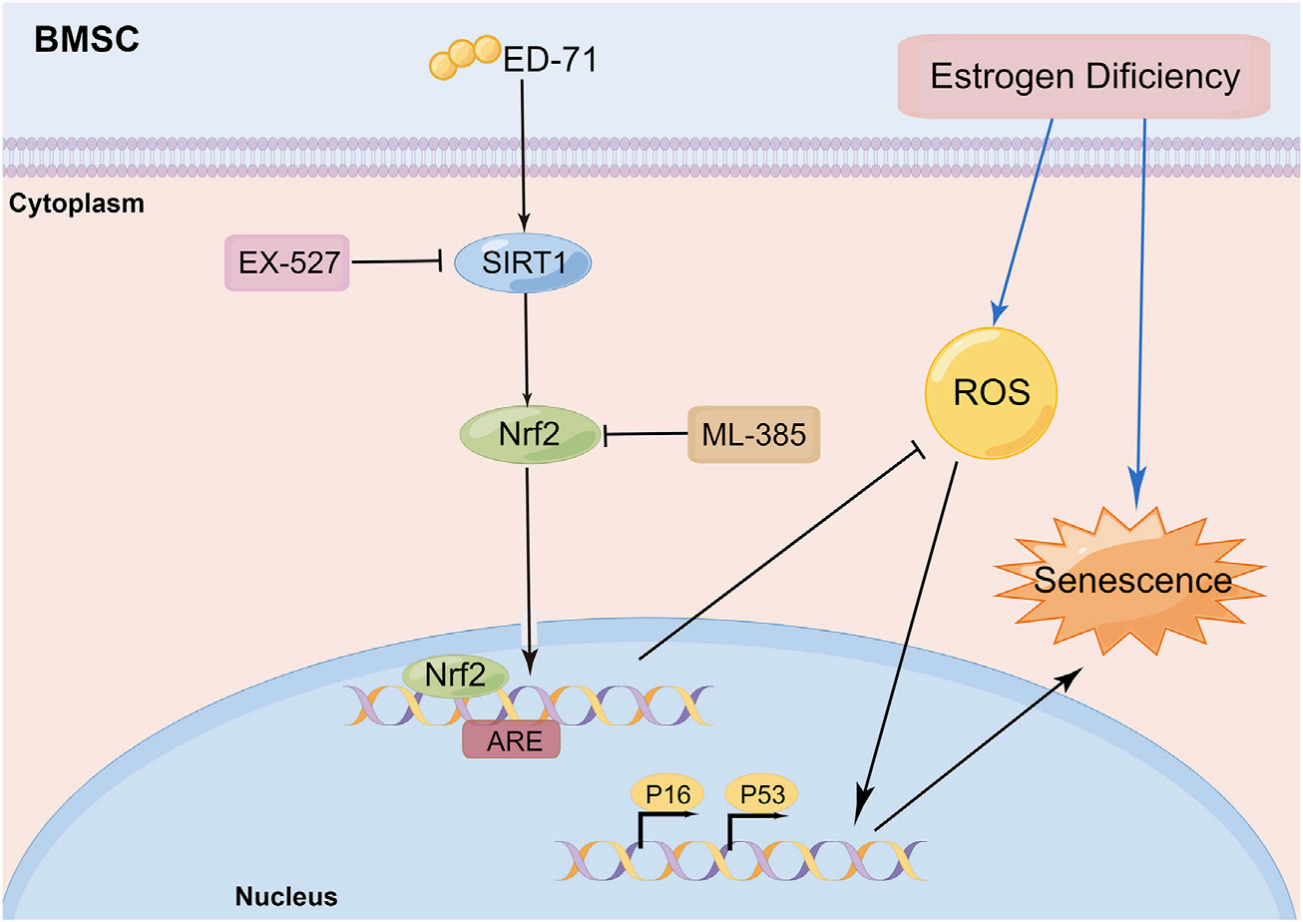
Schematic diagram: ED-71 could inhibit the cell senescence of BMSCs in OVX rats by inhibiting the oxidative stress through the SIRT1-Nrf2 signal (By Figdraw; www.figdraw.com).

In this study, we explored the effect of ED-71 on cell senescence during the process of preventing postmenopausal osteoporosis through *in vivo* and *in vitro* experiments. Our results showed that ED-71 might reduce bone loss in OVX rats by inhibiting the senescence of BMSCs. ED-71 could also protect the BMSCs from damage caused by oxidative stress. In addition, the inhibitory effect of ED-71 on cell senescence might have occurred through the SIRT1-Nrf2 signal (Figure 6). Our results provided a new direction for exploring the mechanism of action of ED-71, that is, cell senescence, an important pathological manifestation of postmenopausal osteoporosis, might be an effective target for ED-71 treatment.

As already known, postmenopausal osteoporosis is caused by estrogen deficiency. Eight-week-old rats were used to establishing an OVX model to simulate this process, which referred to other studies (Wu et al., 2020; Chen et al., 2022). The results showed that the femoral bone mass was significantly decreased in OVX rats, accompanied by an increase in rat weight and fat in bone marrow. This confirmed that our model was successfully established. At the same time, treatment with ED-71 significantly increased the bone volume and the number of trabecular bones, as well as the regeneration of new bone, consistent with a previous report (de Freitas et al., 2011). Interestingly, we found that ED-71 could significantly reduce lipid droplets in the bone marrow of rats. It was suggested that ED-71 might reduce the adipogenic differentiation of BMSCs. Although in a previous study, we have also observed the possible lipid-lowering effect of ED-71 (Lu et al., 2022), whether it is early work or in this study, we have not explored this aspect. Therefore, this may be a direction we study next.

With the decrease in bone mass, the positive expression of aging-related factors increased in the femur area of OVX rats, including the cells of bone marrow and bone surface. A higher expression of aging-related factors was found in the extract of OVX rat bone tissue, which suggested that OVX rats showed a significant aging state. After adding ED-71, the cell senescence caused by OVX was significantly reduced. An increase in cell senescence in postmenopausal osteoporosis has been partially reported (Manolagas, 2010; Wu et al., 2020). Studies have also found that in various osteoporosis models, the senescence of BMSCs was increased and the expression levels of P16 and P53 were raised (Sui et al., 2016). Aging BMSCs show loss of pluripotency, and changes in its differentiation potential, and the dynamic balance between osteogenesis and adipogenesis (Liu et al., 2021). P16 located in the Ink4a gene is strictly controlled by members of the PRC family, and it is a key gene that induces aging (Aguilo et al., 2011). P53, a cell cycle regulatory factor, participates in the function of the cell cycle, apoptosis, and genome stability (Armesilla-Diaz et al., 2009). Senescence-associated acidicβ-galactosidase (SA-β-Gal) is a widely used marker of aging (Itahana et al., 2007; Mohamad Kamal et al., 2020). Next, we extracted the BMSCs from rats in the Sham and OVX groups, and we found that aging cells were significantly increased in BMSCs from OVX rats, which was accompanied by a decrease in osteogenesis. This is consistent with the reported research (Huang T. et al., 2020). More importantly, we found that ED-71 significantly improved the senescence of OVX rat BMSCs, which was proved by β-gal staining, RT-PCR, and Western blot. This further verified our *in vivo* discovery, i.e., regulatiing cell senescence by ED-71 may be an important way to prevent osteoporosis.

An increasing number of studies have shown that active vitamin D is closely related to aging. Evidence shows that the synthesis of vitamin D decreases with age, resulting in aging. Vitamin D participates in maintaining genome stability and telomere length, which is a direct decisive factor for cell senescence (Bima et al., 2021). Active vitamin D could inhibit cell senescence and apoptosis by inhibiting oxidative stress and DNA damage (Chen L. et al., 2019). Active vitamin D deficiency is found to accelerate male reproductive senescence (He et al., 2021). In addition, a study has found that active vitamin D improved age-related osteoporosis through the VDR-EZH2-P16/P19 signaling pathway (Yang et al., 2020). ED-71, a new type of active vitamin D analog, has a better application prospect than traditional vitamin D. Although our previous research has shown that ED-71 might participate in regulating the production of oxidative stress (Huang C. et al., 2020; Zhang et al., 2022), there is no evidence regarding whether ED-71 affects cell senescence. Our results showed that ED-71 could inhibit the senescence of cells induced by OVX, which filled the gap in research on this aspect.

In further research, we found that the addition of ED-71 might play a role in regulating SIRT1 and Nrf2. The inhibitory of SIRT1 and Nrf2 reversed the effect of ED-71 on senescence, which proved that ED-71 could inhibit the senescence of BMSCs by activating SIRT1 and Nrf2 signal. Some studies revealed the connection between SIRT1 and Nrf2. SIRT1 upregulated the downstream signaling pathway of Nrf2 by reducing the acetylation level of Nrf2, thereby improving myocardial ischemia/reperfusion injury (Xu et al., 2021). Galangin can exert anti-oxidant and anti-senescence effects through the SIRT1-PGC-1α/Nrf2 signal (Lee et al., 2022). Our results showed that in ED-71-induced inhibition of BMSC senescence, SIRT1 might act as an upstream signal to regulate Nrf2, which was consistent with previous research. Given the close connection between Nrf2 and oxidative stress, we also detected the effect of ED-71 on oxidative stress. It was consistent with our supposition that ED-71 improved the antioxidant capacity of BMSCs. Therefore, ED-71 might prevent the cell senescence of OVX rat BMSCs by inhibiting oxidative stress, although this requires further experiments to prove.

It is worth mentioning that, we also detected the differences in the osteogenic ability of BMSCs in Sham group and OVX group, and initially explored the effect of ED-71 on the osteogenic differentiation of BMSCs. The osteogenic differentiation ability of BMSCs in OVX group was reduced compared with Sham group, which was consistent with previous study (Huang T. et al., 2020). Although the link between cell senescence and osteogenic differentiation wasn’t verified in this study, it has been confirmed in previous studies. On the one hand, aging BMSCs showed weak osteogenic differentiation potential and enhanced lipogenic differentiation potential (Liu et al., 2015). P16 deletion could ameliorate OVX-induced decrease in osteogenic differentiation of BMSCs (Li et al., 2020). On the other hand, aging BMSCs also produce various tissue-aging stimulating factors, and these bioactive mediators are considered a component of the senescence-associated secretory phenotype (SASP). Molecules of the SASP are secreted into the bone microenvironment by senescent cells, which molecules inhibit the osteogenic differentiation of BMSCs (Xu et al., 2018). We also found that ED-71 could improve the osteogenic differentiation of OVX rat BMSCs. Some studies promoting osteogenic differentiation of BMSCs and improve osteoporosis by regulating cell senescence (Wu et al., 2020; Chen et al., 2022). SIRT1 and Nrf2 have also been found to be associated with the osteogenic differentiation of BMSCs (Wang et al., 2018; Fei et al., 2021). This suggested that ED-71 might regulate the osteogenic differentiation of OVX rat BMSCs by inhibiting cell senescence, which needs to be further verified in future studies.

Taken together, we found that ED-71 inhibited the senescence of OVX rats *in vivo*, and we explored the specific mechanism of ED-71 in regulating the SIRT1-Nrf2 signal *in vitro*. Our study provides a new direction for ED-71 during osteoporosis prevention.

## Data Availability

The raw data supporting the conclusions of this article will be made available by the authors, without undue reservation.
